# Galantamine improves cognition, hippocampal inflammation, and synaptic plasticity impairments induced by lipopolysaccharide in mice

**DOI:** 10.1186/s12974-018-1141-5

**Published:** 2018-04-18

**Authors:** Yi Liu, Yuyun Zhang, Xian Zheng, Tongyong Fang, Xia Yang, Xuan Luo, Anlei Guo, Kelly A. Newell, Xu-Feng Huang, Yinghua Yu

**Affiliations:** 1 Jiangsu Key Laboratory of Immunity and Metabolism, Department of Pathogen Biology and Immunology, Xuzhou Medical University, Xuzhou, 221004 Jiangsu China; 2 Jiangsu Key Laboratory of New Drug Research and Clinical Pharmacy, Xuzhou Medical University, Xuzhou, 221004 Jiangsu China; 3 Illawarra Health and Medical Research Institute, School of Medicine, University of Wollongong, Wollongong, NSW 2522 Australia

**Keywords:** Galantamine, Lipopolysaccharide, NF-κB p65, Microglia, Astrocytes

## Abstract

**Background:**

Neuroinflammation plays an important role in the onset and progression of neurodegenerative diseases such as Alzheimer’s disease. Lipopolysaccharide (LPS, endotoxin) levels are higher in the brains of Alzheimer’s disease patients and are associated with neuroinflammation and cognitive decline, while neural cholinergic signaling controls inflammation. This study aimed to examine the efficacy of galantamine, a clinically approved cholinergic agent, in alleviating LPS-induced neuroinflammation and cognitive decline as well as the associated mechanism.

**Methods:**

Mice were treated with galantamine (4 mg/kg, intraperitoneal injection) for 14 days prior to LPS exposure (intracerebroventricular injection). Cognitive tests were performed, including the Morris water maze and step-through tests. mRNA expression of the microglial marker (CD11b), astrocytic marker (GFAP), and pro-inflammatory cytokines (IL-1β, IL-6, and TNF-α) were examined in the hippocampus by quantitative RT-PCR. The inflammatory signaling molecule, nuclear factor-kappa B (NF-κB p65), and synapse-associated proteins (synaptophysin, SYN, and postsynaptic density protein 95, PSD-95) were examined in the hippocampus by western blotting. Furthermore, NF-κB p65 levels in microglial cells and hippocampal neurons were examined in response to LPS and galantamine.

**Results:**

Galantamine treatment prevented LPS-induced deficits in spatial learning and memory as well as memory acquisition of the passive avoidance response. Galantamine decreased the expression of microglia and astrocyte markers (CD11b and GFAP), pro-inflammatory cytokines (IL-1β, IL-6, and TNF-α), and NF-κB p65 in the hippocampus of LPS-exposed mice. Furthermore, galantamine ameliorated LPS-induced loss of synapse-associated proteins (SYN and PSD-95) in the hippocampus. In the in vitro study, LPS increased NF-κB p65 levels in microglia (BV-2 cells); the supernatant of LPS-stimulated microglia (Mi-sup), but not LPS, decreased the viability of hippocampal neuronal cells (HT-22 cells) and increased NF-κB p65 levels as well as expression of pro-inflammatory cytokines (IL-1β, IL-6) in HT-22 cells. Importantly, galantamine reduced the inflammatory response not only in the BV-2 microglia cell line, but also in the HT-22 hippocampal neuronal cell line.

**Conclusions:**

These findings indicate that galantamine could be a promising treatment to improve endotoxin-induced cognitive decline and neuroinflammation in neurodegenerative diseases.

## Background

Neuroinflammation has been considered as a critical driver of cognitive deficits associated with several neurodegenerative diseases, such as Alzheimer’s disease, diabetic encephalopathy, and amyotrophic lateral sclerosis [1–3]. Hippocampal inflammation has been reported to contribute to postoperative cognitive decline and Alzheimer’s disease [4, 5]. Lipopolysaccharide (LPS, endotoxin), a cell wall component of gram-negative bacteria, has been shown to induce hippocampal neuroinflammation and cognitive impairment and is widely used in preclinical models to model the postoperative cognitive dysfunction (POCD) [6–9]. The levels of LPS and its related gram-negative *Escherichia coli* bacteria were reported to be higher in the brains of subjects with Alzheimer’s disease [10]. However, the mechanisms underlying the role of LPS in deleterious neuroinflammation in cognitive decline are poorly understood.

Neuroglial cells are essential for the maintenance of brain homeostasis; however, overactivated neuroglial cells, such as microglia and astrocytes, contribute to neuroinflammation and neurodegenerative disorders [11]. Enhanced expression of CD11b, a β-integrin marker of microglia, represents microglial activation during neurodegenerative inflammation [12]. Activation of the nuclear factor-kappa B (NF-κB) in microglia promotes gene expression of pro-inflammatory cytokines, such as interleukin 1β and 6 (IL-1β, IL-6) and tumor necrosis factor-α (TNF-α), which contribute to neurodegenerative disorders [13, 14]. Glial fibrillary acidic protein (GFAP) is a marker for astrogliosis [15]. Recently, it has been reported that LPS activates pro-inflammatory microglia, while astrocytes existing alone in culture cannot respond to LPS, as rodent astrocytes lack TLR4 and MYD88 downstream signaling components required for LPS activation [11].

Cognitive impairment is associated with synaptic abnormalities in the hippocampus [16]. Pre-synaptic and post-synaptic proteins such as synaptophysin (SYN) and postsynaptic density protein (PSD-95), respectively, play important roles in synaptic plasticity and cognitive function [17]. SYN protein levels are lower in oldest-aged individuals with dementia [18]. PSD-95 levels are decreased in brains of patients with Alzheimer’s disease compared to controls [19]. It has been shown that there is a direct interaction between neuroinflammation and SYN as well as PSD-95 in newborn neurons [20, 21]. Co-activation of proinflammatory cytokines and cytotoxic products in neuroinflammation processes are destructive to neurons by altering synaptic proteins [21]. Therefore, the neuroinflammatory response may induce the loss of synaptic proteins and cognitive deficits in neurodegenerative illness.

Galantamine is an alkaloid obtained from the bulbs and flowers of the Caucasian snowdrop (Vornonov’s snowdrop) and *Lycoris radiate* (Red Spider Lily)-related species [22]. Pharmacokinetic studies demonstrate that galantamine can quickly cross the blood-brain barrier and remain in brain regions, such as the hippocampus, for an extended time (biological half-life is 5–7 h) [23, 24]. Galantamine, a centrally acting acetyl-cholinesterase (AChE) inhibitor, is used safely in elderly Alzheimer’s patients to improve cognitive function [25]. Galantamine increases the release of Ach in the hippocampus, which is essential for learning and memory [26, 27]. Recent studies have demonstrated that galantamine has significant anti-inflammatory effects in vitro and in vivo. For example, galantamine and nicotine have a synergistic effect on inhibition of TNF-α release and cellular activation in a cultured microglia model of HIV-associated dementia [28]. In mice, galantamine decreases serum TNF-α and IL-6 concentrations and enhances the survival of mice following an intraperitoneal injection of lethal and inflammatory doses of LPS [29]. However, the ability of galantamine to prevent LPS-induced cognitive decline is yet to be reported and the mechanisms underlying its cognitive enhancing and anti-neuroinflammation properties remained to be clarified. In the present study, we examined if galantamine could improve cognitive behavior and neuronal morphology in a neuroinflammation mouse model developed using intracerebroventricular (ICV) administration of LPS. To further investigate the underlying mechanisms, the hippocampal synaptic proteins (SYN and PSD-95), neuroglial cell markers (CD11b and GFAP), the pro-inflammatory transcription factor, NF-κB p65, and cytokines (IL-1β, TNF-α and IL-6) were also examined in the hippocampus. Moreover, to investigate the precise cell and molecular mechanism of LPS-induced neuroinflammation and the specific anti-inflammatory target of galantamine, the NF-κB p65 levels in microglia cells and hippocampal neurons were examined in response to LPS and galantamine.

## Methods

### Animals

Male Kunming mice (6 weeks old weighing 18–22 g) were used in the present study. The mice were obtained from the Experimental Animal Center of Xuzhou Medical University (Xuzhou, China, SCXK(Su)2015-0009). The mice were housed with ad libitum access to food and water under temperature- and humidity-controlled conditions with a 12-h light/dark cycle. All procedures were approved by the Animal Ethics Committee, Xuzhou Medical University, China, and complied with the National Institutes of Health Guidelines for the Care and Use of Laboratory Animals.

### Experimental protocol

The mice were randomized into four groups (*n* = 13 per group): (1) the control group—vehicle treatment and ICV injection of saline; (2) the LPS group—vehicle treatment and ICV injection of LPS; (3) the galantamine treatment group (LPS + galantamine)—galantamine followed by LPS ICV injection; (4) the galantamine control group (galantamine)—galantamine followed by saline ICV injection. Mice were injected with LPS (8 μg/μl in 3 μl, ICV) or saline into the lateral ventricle (AP − 0.46, ML − 1.0, and DV − 1.8) as previously described [30]. Mice were continuously treated with galantamine (4 mg/kg, ip injection) or vehicle treatment for 14 days starting 1 day before the LPS injection. This dosage of galantamine is in accordance with previous studies showing that it inhibits peripheral inflammation in rodents [31, 32]. Galantamine hydrobromide was purchased from Shanghai Xudong Haipu Pharmaceutical Co., Ltd. (Shanghai, China). LPS (*Escherichia coli*, serotype 0127: B8) was purchased from Sigma (St. Louis, MO, USA). Similar to previous studies [8, 33], behavioral tests were assessed 5 days after the LPS injection (10 mice per group). Within 1 day after the final test, mice were sacrificed and the hippocampus were collected: the left hippocampus was used to measure CD11b, GFAP, IL-1β, IL-6, and TNF-α by RT-PCR (*n* = 5 per group); the right hippocampus was used to measure NF-κB p65, SYN, and PSD-95 by western blot (*n* = 5 per group); neuronal morphometry by Golgi staining was also performed on the remaining three of each group that did not undergo behavioral testing.

### Behavioral tests

#### Morris water maze test

As previously described [34], the Morris water maze (MWM) test included four consecutive daily training trials and a probe trial on the fifth day. The test was performed in a circular pool (120 cm in diameter and 60 cm in height) filled with water. The pool was divided into four quadrants with an escape platform (10 cm in diameter) hidden in water in the center of one quadrant. During training trials, the time that the mice spent to reach the hidden platform was recorded as escape latency. On the probe trial, the platform was removed. The time taken to cross the former platform location was recorded to indicate the degree of memory consolidation.

#### Step-through test

The step-through test was performed to examine memory acquisition as previously described [35, 36]. Briefly, the test included a training trial on the first day and retention trial on the second day. The apparatus consisted of a compartment with two chambers (a light chamber equipped with an illuminator and a dark chamber) and an interconnecting semicircular door. During the training trial, the mice were placed in the light chamber for 3 min. After the door opened, the mice moved to the dark chamber and received a footshock for 1 s. During the retention trial, the number of mistakes and the time that the mice took before initially entering the dark chamber (the step-through latency) were recorded for 5 min.

### Reverse transcriptase-PCR (RT-PCR)

After total RNA was extracted using TRI reagent (Sigma-Aldrich, MO, USA), cDNA was synthesized by a High-Capacity RNA-to-cDNA kit (Sigma-Aldrich, MO, USA). The primers for CD11b, GFAP, IL-1β, IL-6, TNF-α, and the housekeeping gene β-actin (Sangon Biotech Co. Ltd., Shanghai, China) are listed in Table 1. Agarose gel electrophoresis was used to separate amplified products followed by a UV trans-illuminator and photography for visualization. Duplicate reaction was performed to verify reproducibility. The values obtained for the target gene expression were normalized to β-actin and quantified relative to the expression in control samples. The products were analyzed by densitometry using the Quantity One 1-D analysis software (BioRad, Hercules, CA, USA).

**Table 1:** Primer sequences for RT-PCR analysis

Target mRNA sequences	Primer sequence	Annealing Tm (°C)
GFAP	5′-AAGCAGATGAAGCCACCCTG-3′ 5′-GTCTGCACG-GGAATGGTGAT-3′	59
CD11b	5′-CAGATCAACAATGTGACCGTATGGG-3′ 5′-CATCATGTCCTTGTACTGCCGCTTG-3′	66
IL-1β	5′-TTGACGGACCCCAAAAGATG-3′ 5′-AGAAGGTGCTCATGTCCTCA-3′	59
IL-6	5′-CGGAGAGGAGACTTCACAGAG-3′ 5′-CATTTCCACGATTTCCCAGA-3′	59
TNF-α	5′-TATGGCTCAGGGTCCAACTC-3′ 5′-GGAAAGCCCATTTGAGTCCT-3′	59
β-actin	5′-ATGGTCACGCACGATTTCCC-3′ 5′-GAGACCTTCAACACCCCAGC-3′	59
IL-6	5′-AGACTTCCATCCAGTTGCCTTCTTG-3′ 5′-CATGTGTAATTAAGCCTCCGACTTGTG-3′	59
IL-1β	5′-TTCAGGCAGGCAGTATCACTCATTG-3′ 5′-ACACCAGCAGGTTATCATCATCATCC-3′	59
TNF-α	5′-GCGACGTGGAACTGGCAGAAG-3′ 5′-GAATGAGAAGAGGCTGAGACATAGGC-3′	59
GAPDH	5′-GGTGAAGGTCGGTGTGAACG-3′ 5′-CTCGCTCCTGGAAGATGGTG-3′	60

### Quantitative real-time PCR (qPCR)

Total RNA was isolated from hippocampal neuronal cells (HT-22 cells), using the TRIzol® reagent (Invitrogen Co., Carlsbad, CA, USA) according to the manufacturer’s instructions. The concentration and purity of total RNA were determined using NanoDrop 1000 (Thermo Scientific). The Rever TraAce qPCR RT kit (TOYOBO) was used for the reverse transcription of total RNA to cDNA. In real-time experiments, cDNA was analyzed in duplicate using 0.2 μmol/L specific primers (Table 1) and 1× LightCycler 480 SYBR green I Master (Roche Applied Science, Germany) in a total volume of 10 μl. GAPDH was used as an endogenous control. PCRs were carried out in a Light Cycle 480 (Roche Applied Science, Germany) using a thermal profile of 10 min at 95 °C followed by 50 cycles of 15 s at 95 °C, 30 s at 60 °C, a melting curve of 15 s at 95 °C, 60 s at 1 min, heating to 95 °C, and cooling for 30 s at 4 °C. The results were analyzed using LightCycler 480 software (version 1.5, Roche Applied Science, Germany). Relative levels of mRNA were analyzed using the ∆∆Ct method.

### Western blotting

Frozen hippocampus samples were cut into small pieces and homogenized in ice-cold extraction buffer [37]. Homogenates were centrifuged at 12,000×*g* for 15 min at 4 °C. With the homogenates restored by agitation, the centrifugation was repeated and the final supernatants obtained. In contrast to frozen hippocampus tissues, BV-2 cells and HT-22 cells were washed twice with PBS (pH 7.4) and centrifuged at 1000 rpm for 5 min. Cell pellets were lysed in an ice-cold extraction buffer [37]. Cell lysates were centrifuged at 12,000×*g* for 15 min at 4 °C. The supernatant was collected and used for further protein concentration analyses. Nuclear extracts only for NF-κB p65 analysis were obtained with a nuclear isolation kit (Beyotime Institute of Biotechnology, Shanghai, China). Protein concentrations were determined using a Pierce BCA Protein Assay Kit (Beyotime Institute of Biotechnology, Shanghai, China). Equal amounts of protein were separated by 10% SDS-polyacrylamide gel electrophoresis and transferred onto nitrocellulose membranes. Membranes were blocked via 5% skim milk powder in Tris-buffered saline including 0.05% (*v*/*v*) Tween 20 (TBST) for 2 h at 25 °C and then incubated overnight with the primary antibodies to NF-κB p65 (1:1000), SYN (1:1000), PSD-95 (1:2000) (Cell Signaling Technology, Inc., Danvers, MA, USA), and β-actin (1:1000; ZSGB-BIO, Beijing, China). Membranes were washed thrice with TBST over 15 min and incubated with secondary antibodies (ZSGB-BIO, Beijing, China) in 5% skim milk powder in TBST. The membranes were exposed to BCIP/NBT alkaline phosphatase color developing reagent (Beyotime Institute of Biotechnology, Shanghai, China) for 15 min. Bands corresponding to the proteins of interest were scanned and band density analyzed using the Quantity One automatic imaging analysis system (Bio-Rad). All quantitative analyses were normalized to β-actin, as per our previous studies [38, 39].

### Golgi staining

Golgi staining was used to assess neuronal morphometry, as is widely used in the research of neurodegenerative disorders [39]. The mice brains (*n* = 3 per group) were stained using a Golgi-staining kit following the manufacturer’s protocol (Genmed Scientifics Inc., Arlington, MA, USA). Golgi-stained brains were sectioned using a vibratome (LEICA VT 1000S) at room temperature (100 μm). The sections were visualized under an upright microscope by two independent researchers blinded to the experiment design (Olympus BX43F microscope, Tokyo, Japan). For spine analysis, the dendrites in 20–30-μm lengths of the three tertiary segments were used to measure dendritic spine density. Three neuronal cells per brain slice and three brain slices per animal were chosen for spine quantitative analysis [40].

### Cell culture and treatment

BV-2 cells and HT-22 cells were obtained from Jiangsu Key Laboratory of New Drug Research and Clinical Pharmacy, Xuzhou Medical University (Xuzhou, China). Dulbecco’s modified Eagle’s medium (DMEM) and fetal bovine serum (FBS) were purchased from Gibco (Grand Island, NY, USA). The cells were grown in DMEM containing 10% FBS, 100 U/mL penicillin, and 100 U/mL streptomycin at 37 °C in a humidified atmosphere of 95% air and 5% CO_2_. Galantamine was from ApexBIO (Cat NO: A3423). LPS (*Escherichia coli*, serotype 0127: B8) was obtained from Sigma (St. Louis, MO). For experiments indicated below, BV-2 cells were exposed to LPS (1 μg/ml) or pretreated with galantamine (10 μM) for 24 h before LPS exposure. Hippocampal neuronal cells (HT-22 cells) were exposed to LPS (1 μg/ml), the supernatant of LPS-stimulated microglia (Mi-sup) or pretreated with galantamine (10 μM) for 24 h prior to Mi-sup exposure.

### Cell viability assay

BV-2 cells and HT-22 cells were seeded and grown for 24 h in 96-well plates at a density of 5 × 10^3^ cells/well. The cells were exposed to LPS (1 μg/mL) or Mi-sup for 24 h. After the well was exposed to 3-(4,5-dimethylthiazol-2-yl)-2,5-dipheyltetrazolium bromide (MTT) (0.5 mg/mL) for 4 h, the MTT was gently removed by aspiration. The formazan crystals were dissolved in dimethyl sulfoxide and the absorbance read at 550 nm by a microplate reader. Cell viability was expressed as a percentage of untreated control cells.

### Immunofluorescent staining

BV-2 cells were seeded on glass cover slips in a 24-well plate at 1 × 10^4^ cells/well and cultured with the Mi-sup at 37 °C for 24 h. After treatment, BV-2 cells were washed three times with PBS, immediately fixed in 4% paraformaldehyde for 30 min and permeabilized with 0.5% Triton X-100 for 15 min. The cells were incubated with primary antibodies against NF-κB p65 (1:50 dilution) overnight at 4 °C. Cells were then washed three times with PBS and incubated with FITC-conjugated goat anti-rabbit secondary antibody (1:200 dilution) for 1 h at room temperature. Cells were washed three times in PBS and stained with DAPI (10 μg/mL) for nuclear identification. The image was visualized and captured by a microscope (Olympus X51W, Olympus Microsystems).

### Statistical analysis

The statistical comparisons were analyzed by two-way repeated measures ANOVA, two way ANOVA, or one-way ANOVA followed by LSD post hoc comparisons for data with equal variances, or by Dunnett’s T3 for data with unequal variances. All analyses were performed using SPSS16.0. *P* values of less than 0.05 were regarded as statistically significant. All values are expressed as mean ± SEM.

## Results

### Galantamine improves cognition in LPS-exposed mice

**Fig. 1:**
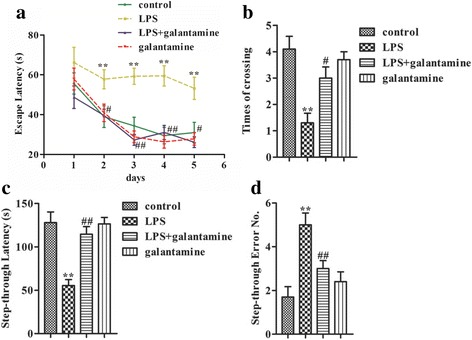
Galantamine improved the cognition in LPS-exposed mice. **a** Escape latency during the training and the probe sessions in MWM test. **b** Times of crossing the former platform location during the probe trial in MWM test. Step-through latency (**c**) and error number (**d**) in the step-through test. Data are reported as mean ± SEM (*n* = 10). ^*^P < 0.05, ^**^P < 0.01, as compared with the control group; ^#^*P* < 0.05, ^##^*P* < 0.01, as compared with the LPS group

Spatial learning and memory along with memory acquisition of the passive avoidance response were measured in response to LPS exposure and galantamine treatment by the MWM test and the step-through test respectively. Two-way repeated measures ANOVA showed that the escape latency was significantly different among groups (F_3,36_ = 13.659, *P* < 0.001), and there was a significant effect of test time (F_4,36_ = 20.543, *P* < 0.001). There was no interaction effect of groups and test time (F_12,36_ = 1.307, *P* = 0.221). During training trials from days 2–4, LPS-exposed mice showed an increase in escape latency compared to the control mice (Fig. 1a, all *P* < 0.01). However, the escape latency during training days 2–4 was significantly decreased in the LPS + galantamine group compared to the LPS group (all *P* < 0.05). There was no significant difference in escape latency between the galantamine control and control groups (*P* > 0.05). In the probe trial on day 5, mice in the LPS group showed increased escape latency (*P* < 0.01, Fig. 1a) and decreased frequency of crossing the former platform location (*P* < 0.01, Fig. 1b) compared with mice form the control group, indicating that spatial memory related to the visual cues was decreased in LPS-treated mice. The LPS + galantamine group showed a significant decrease in the escape latency (*P* < 0.05, Fig. 1a) and increase in frequency of crossing the former platform location compared with the LPS group (*P* < 0.05, Fig. 1b). The galantamine control group performed similarly to the control group (*P* > 0.05; Fig. 1), indicating that galantamine per se did not influence the spatial learning and memory of mice in the control group.

In the step-through test, two-way ANOVA-indicated LPS (F_1,36_ = 21.432, *P* < 0.001) and galantamine (F_1,36_ = 9.983, *P* = 0.003) both showed effects on the step-through latency, and there was an interaction effect between LPS and galantamine (F_1,36_ = 11.122, *P* = 0.002). LPS injection decreased the step-through latency compared to the control group (*P* < 0.01), while the step-through latency of the LPS + galantamine treatment group was increased compared to the LPS group (*P* < 0.01, Fig. 1c). In addition, two-way ANOVA indicated that the number of mistakes was significantly affected by LPS (F_1,36_ = 17.894, *P* < 0.001), but not galantamine (F_1,36_ = 1.988, *P* = 0.167), and there was an interaction effect between LPS and galantamine (F_1,36_ = 8.576, *P* = 0.006). The number of mistakes made by the LPS group significantly increased compared to the control group (*P* < 0.01, Fig. 1d), while the number of errors in the LPS + galantamine group was significantly decreased compared to the LPS group (*P* < 0.01). There were no statistical differences between the control and galantamine control groups in step-through latency to initial entry or in the number of mistakes (*P* > 0.05).

### Galantamine prevents the upregulation of CD11b and GFAP mRNA levels in the hippocampus of the LPS-exposed mice

**Fig. 2:**
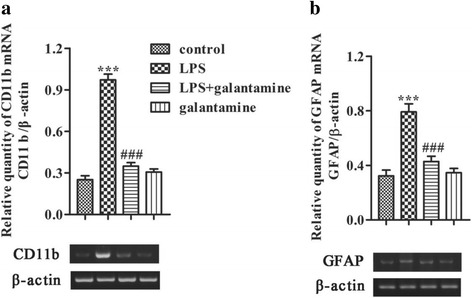
Galantamine prevented the activation of neuroglia cells in the hippocampus of the LPS-exposed mice. The mRNA levels of CD11b (**a**) and GFAP (**b**) in the hippocampus of mice were examined by reverse transcriptase-PCR (RT-PCR). The level of each mRNA was normalized to the mRNA level of β-actin as an endogenous control. Data are reported as mean ± SEM (*n* = 5). ^**^P < 0.01, ^***^P < 0.001, as compared with the control group; ^##^*P* < 0.01, ^###^*P* < 0.001, as compared with the LPS group

Activation of neuroglia is implicated in the pathogenesis of memory decline in neuroinflammatory and neurodegenerative diseases [12, 41, 42]. We examined the mRNA level of the microglia and astrocyte markers, CD11b and GFAP, in the hippocampus in response to LPS and galantamine. Two-way ANOVA showed that LPS and galantamine affected CD11b mRNA expression (LPS, F_1,16_ = 158.084, *P* < 0.001; galantamine, F_1,16_ = 87.914, *P* = 0.003) and GFAP mRNA expression (LPS, F_1,16_ = 39.174, *P* < 0.001; galantamine, F_1,16_ = 14.931, *P* = 0.001). There were also significant interaction effects between LPS and galantamine on CD11b mRNA (F_1,16_ = 125.232, *P* < 0.001) and GFAP mRNA (F_1,16_ = 19.342, *P* < 0.001). LPS significantly increased CD11b mRNA expression (*P* < 0.001, Fig. 2a) and GFAP mRNA expression (*P* < 0.001, Fig. 2b) in the hippocampus compared to the control group. Galantamine treatment (LPS + galantamine group) significantly prevented the elevation of CD11b and GFAP mRNA induced by LPS (both *P* < 0.001; Fig. 2). These results suggest that galantamine prevents microglial and astrocyte activation in the hippocampus of mice following LPS exposure.

### Galantamine prevents the upregulation of NF-κB p65, and IL-1β, IL-6, and TNF-α mRNA levels in the hippocampus of LPS-exposed mice

**Fig. 3:**
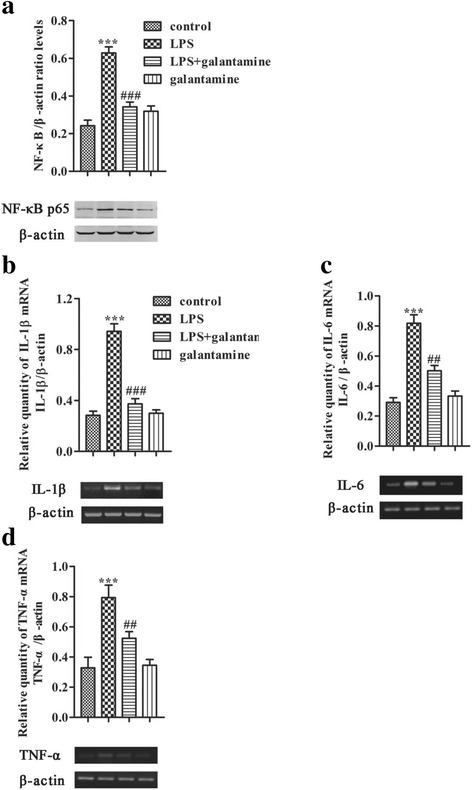
Galantamine attenuated neuroinflammation in the hippocampus of LPS-exposed mice. The protein level of NF-κB p65 was examined by western blot (**a**). The mRNA levels of IL-1β (**b**), IL-6 (**c**), and TNF-α (**d**) in the hippocampus of mice were examined by reverse transcriptase-PCR (RT-PCR). The level of each mRNA was normalized to the mRNA level of β-actin as an endogenous control. Data are reported as mean ± SEM (*n* = 5). ^**^P < 0.01, ^***^P < 0.001, as compared with the control group; ^##^*P* < 0.01, ^###^*P* < 0.001, as compared with the LPS group

To further determine whether changes in neuroglia could be associated with neuroinflammation, we measured the protein levels of NF-κB p65, transcription factors in inflammatory signaling. Two-way ANOVA showed that the levels of NF-κB p65 were affected by LPS (F_1,16_ = 49.842, *P* < 0.001), galantamine (F_1,16_ = 12.964, *P* = 0.002) and the interaction between LPS and galantamine (F_1,16_ = 38.906, *P* < 0.001). LPS exposure significantly increased NF-κB p65 in the hippocampus compared to the control group (*P* < 0.001; Fig. 3a). Galantamine treatment significantly prevented the LPS-induced NF-κB p65 elevation in mice (*P* < 0.001; Fig. 3a). There were no significant differences in the levels of NF-κB p65 between the control and galantamine control groups (*P* > 0.05; Fig. 3a). These results suggest that galantamine prevents LPS-induced activation of NF-κB p65, a transcription factor that plays crucial roles in neuroinflammation.

Next, we examined if galantamine could decrease the mRNA expression of the proinflammatory cytokines, IL-1β, IL-6, and TNF-α, which are transcribed by NF-κB. Two-way ANOVAs indicated that the mRNA expression of pro-inflammatory factors were affected by LPS and galantamine, including IL-1β (LPS, F_1,16_ = 76.534, *P* < 0.001; galantamine, F_1,16_ = 43.762, *P* < 0.001), IL-6 (LPS, F_1,16_ = 73.814, *P* < 0.001; galantamine, F_1,16_ = 11.453, *P* = 0.004), and TNF-α (LPS, F_1,16_ = 27.326, *P* < 0.001; galantamine, F_1,16_ = 4.205, *P* = 0.057). LPS and galantamine had significant interaction effects on the mRNA expression of IL-1β (F_1,16_ = 48.854, *P* < 0.001), IL-6 (F_1,16_ = 19.740, *P* < 0.001), and TNF-α (F_1,16_ = 5.427, *P* = 0.033). LPS exposure significantly increased the mRNA expression of IL-1β (*P* < 0.001; Fig. 3b), IL-6 (*P* < 0.001; Fig. 3c), and TNF-α (*P* < 0.001; Fig. 3d) in the hippocampus compared to the control group. Galantamine treatment significantly prevented the LPS-induced upregulation of these pro-inflammatory cytokine transcripts (IL-1β: *P* < 0.001, IL-6: *P* < 0.01, TNF-α: *P* < 0.01; Fig. 3). There were no significant differences in IL-1β, IL-6, and TNF-α mRNA expression between the control and galantamine control groups (all *P* > 0.05; Fig. 3).

### Galantamine prevents the reduction of synapse-associated proteins and neurodegeneration in the hippocampus of LPS-exposed mice

**Fig. 4:**
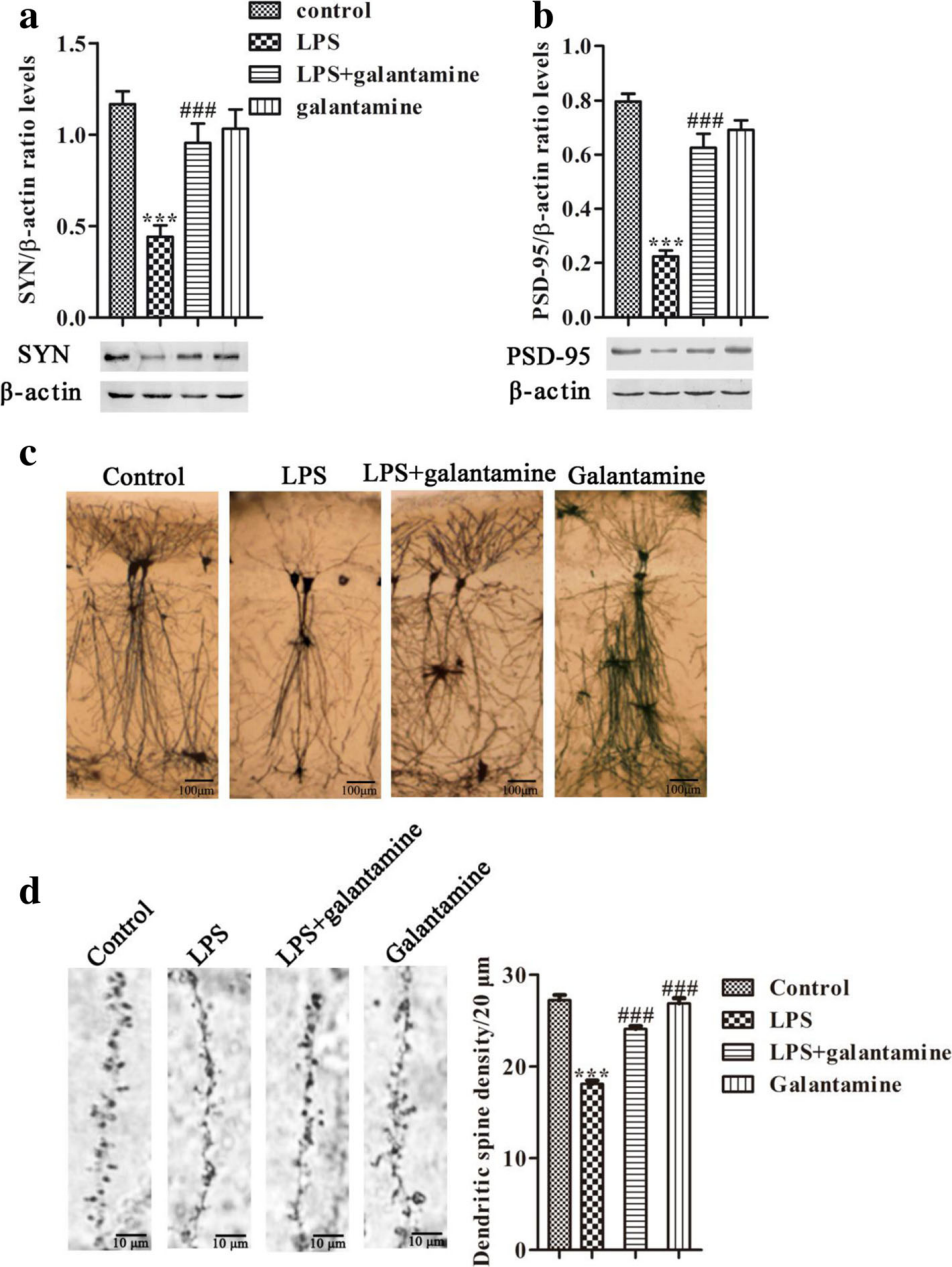
Galantamine prevented the reduction of synapse-associated proteins and neurodegeneration in the hippocampus of LPS-exposed mice. The protein levels of SYN (**a**) and PSD-95 (**b**) were examined by western blot. Data are reported as mean ± SEM (*n* = 5). Golgi silver-stained neurons in the hippocampal CA1 subfield (**c**). Representative images are shown (*n* = 3). Bar = 100 μm. **d** Representative dendritic spine and dendritic spine density at the CA1 hippocampal region (*n* = 3). Bar = 10 μm. ^**^P < 0.01, ^***^P < 0.001, as compared with the control group; ^##^*P* < 0.01, ^###^*P* < 0.001, as compared with the LPS group

There is growing evidence that synaptic dysfunction and degeneration contribute to the deterioration of memory performance [43, 44]. In the two-way ANOVA, we found that LPS and galantamine significantly affected synaptic proteins including SYN (LPS, F = 21.010, *P* < 0.001; galantamine, F_1,16_ = 4.713, *P* = 0.045) and PSD-95 (LPS, F_1,16_ = 78.366, *P* < 0.001; galantamine, F_1,16_ = 16.830, *P* = 0.001). There were also interaction effects between LPS and galantamine on SYN (F_1,16_ = 13.690, *P* = 0.002) and PSD-95 (F_1,16_ = 49.130, *P* < 0.001). Western blot analyses showed a reduction of the pre-synaptic protein SYN (*P* < 0.001, Fig. 4a) and post-synaptic protein PSD-95 (*P* < 0.001, Fig. 4b) in the hippocampus of mice treated with LPS. Treatment with galantamine significantly attenuated the decline of both SYN and PSD-95 proteins in the hippocampus of LPS mice compared to LPS-treated mice (*P* < 0.001, Fig. 4a b). In serial coronal sections of the hippocampus, neurons in the LPS mice had less dendritic branching, reduced branch length, and decreased complexity of dendritic trees in the hippocampal CA1 subfield compared to the other groups, which was reversed by galantamine treatment (Fig. 4c). In addition, dendritic spine density was also significantly decreased in LPS mice, which was restored by galantamine (*P* < 0.001, Fig. 4d).

**Fig. 5:**
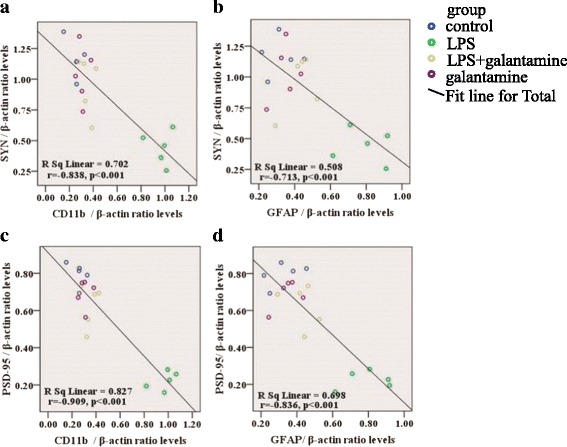
Correlations between synapse-associated proteins and glia markers. There were negative correlations between synapse-associated proteins (SYN and PSD-95) and glia markers (CD11b and GFAP) (**a**–**d**)

The activation of microglia and astrocytes has been found to be involved in synaptic plasticity in mice [45]. Thus, we examined the correlations between the synaptic proteins and the levels of CD11b and GFAP mRNA in the hippocampus. Pearson correlation analysis showed that there was a remarkable negative correlation between CD11b and GFAP mRNA levels with SYN expression in the hippocampus (CD11b: *r* = − 0.838, *P* < 0.001, Fig. 5a; GFAP: *r* = − 0.713, *P* < 0.001, Fig. 5b). Furthermore, there were significant negative correlations between CD11b and GFAP mRNA levels with PSD-95 expression in the hippocampus (CD11b: *r* = − 0.909, *P* < 0.001 Fig. 5c; GFAP: *r* = − 0.836, *P* < 0.001, Fig. 5d).

### Mi-sup rather than LPS decreases HT-22 cell viability and increases the levels of NF-κB p65 in HT-22 cells, and LPS increases the levels of NF-κB p65 in BV-2 cells

**Fig. 6:**
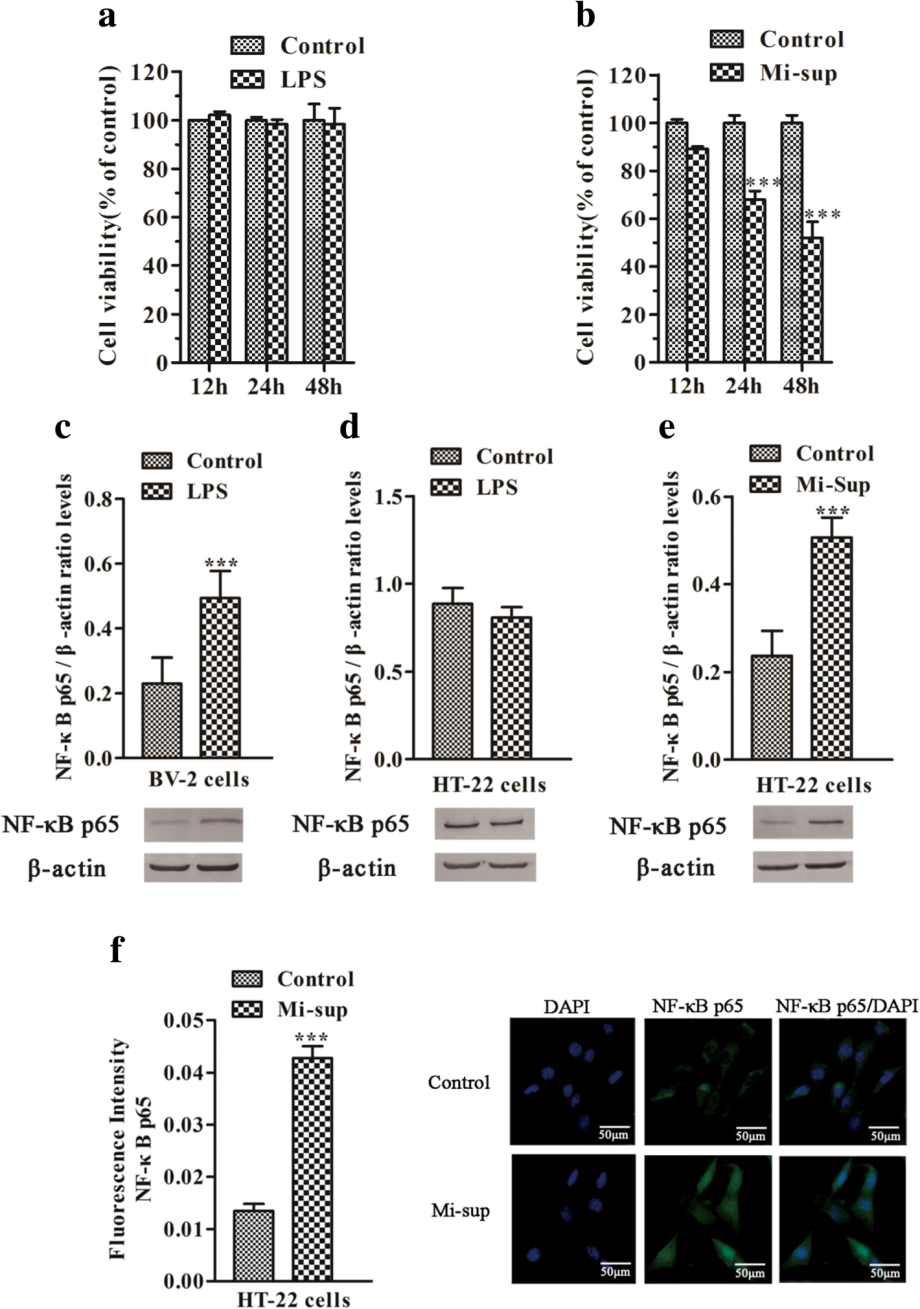
Mi-sup but not LPS decreased the viability and activated NF-κB p65 of HT-22 cells. MTT assays were performed to detect the cell viability in HT-22 cells treated with LPS (**a**) or Mi-sup (**b**) for 12, 24, or 48 h. Results are expressed relative to control and are presented as means ± SEM of three independent experiments (each performed in triplicate). The levels of NF-κB p65 in LPS-exposed BV-2 cells (**c**) and LPS-exposed HT-22 cells (**d**) were determined by western blot. HT-22 cells were exposed to Mi-sup for 24 h, and then the activation of NF-κB p65 was determined by western blot (**e**) and immunofluorescence staining (**f**). Data are presented as means ± SEM (*n* = 5). ^*^P < 0.05, ^**^P < 0.01,^***^P < 0.001 vs control group

The cytotoxicity of LPS on cell viability of HT-22 cells (a hippocampal neuronal cell line) and BV-2 cells (a microglia-derived cell line) were determined by the MTT assay 12, 24, and 48 h after LPS or Mi-sup exposure. Two-way repeated measures ANOVA indicated that LPS and time did not significantly influence HT-22 cell viability (F_1,4_ = 0.007, *P* = 0.938; F_2,4_ = 0.166, *P* = 0.717, Fig. 6a). There was no interaction effect between LPS and time (F_2,4_ = 0.166, *P* = 0.717). However, the HT-22 cell viability was significantly affected by Mi-sup exposure and time and there was an interaction between two factors (F_1,4_ = 257.606, *P* < 0.001; F_2,4_ = 9.487, *P* = 0.008; F_2,4_ = 9.487, *P* = 0.008). After 24 and 48 h of Mi-sup exposure, HT-22 cell viability was significantly decreased compared with the control group (both *P* < 0.001, Fig. 6b). Therefore, LPS did not directly decrease HT-22 viability, but the supernatant of LPS-stimulated BV-2 microglia decreased HT-22 cell viability.

Next, the pro-inflammatory signaling molecule, NF-κB p65, was examined by western blot in BV-2 cells and HT-22 cells in response to LPS or Mi-sup. LPS significantly increased the levels of NF-κB p65 in BV-2 cells (*P* < 0.001, Fig. 6c), but not in HT-22 cells (*P* > 0.05, Fig. 6d) compared to the control group. However, in HT-22 cells, the levels of NF-κB p65 were significantly increased by Mi-sup (*P* < 0.001, Fig. 6e). We further confirmed that immunofluorescence of NF-κB p65 was increased in HT-22 cells in response to Mi-sup exposure (*P* < 0.001, Fig. 6f). These observations suggest that LPS activates NF-κB signaling on HT-22 neurons indirectly via activating BV-2 microglia first.

### Galantamine prevents the increase of NF-κB p65 in BV-2 cells and HT-22 cells

**Fig. 7:**
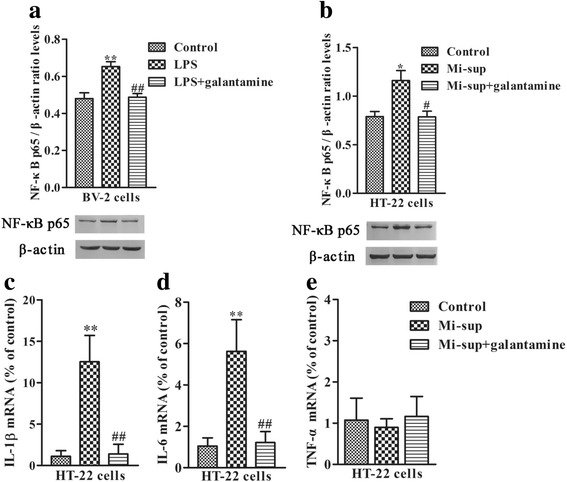
Galantamine prevented the increase of NF-κB p65 in LPS-exposed BV-2 cells, and the increase of NF-κB p65, IL-1β, and IL-6 in Mi-sup-exposed HT-22 cells. The protein level of NF-κB p65 was examined by western blot (**a** **b**), and the mRNA expression of IL-1β, IL-6, and TNF-α were examined by real-time PCR (**c**-**e**). Data are reported as mean ± SEM (*n* = 5). ^*^P < 0.05, ^**^P < 0.01, as compared with the control group; ^#^*P* < 0.05, ^##^*P* < 0.01, as compared with the LPS group or Mi-sup group

To further explore the anti-neuroinflammatory mechanism of galantamine, we examined whether galantamine can prevent the increase of NF-κB p65 in LPS-exposed BV-2 cells and Mi-sup-exposed HT-22 cells. Here we found that galantamine treatment (10 μM) resulted in a decrease of NF-κB p65 in BV-2 cells compared to the LPS group (*P* < 0.01, Fig. 7a) and in HT-22 cells versus the Mi-sup group (*P* < 0.05, Fig. 7b). Furthermore, Mi-sup increased pro-inflammatory cytokines IL-1β and IL-6 mRNA, but not TNF-α mRNA expression, in HT-22 cells, while galantamine inhibited the inflammation induced by Mi-sup (Fig. 7c–e).

## Discussion

This study showed that galantamine prevented LPS-induced cognitive deficits, including deficits in spatial learning and memory as well as memory acquisition of the passive avoidance response in mice. Of particular significance were the findings that galantamine inhibited the gliosis, activation of microglia and astrocyte, the expression of NF-κB p65 and pro-inflammatory cytokines in the hippocampus of LPS-exposed mice. Consequently, galantamine ameliorated LPS-induced reductions in hippocampal synaptic proteins, and dendritic branching and spine density. Further, LPS induced neuroinflammation by directly activating the microglia, whereas galantamine reduced the inflammatory response not only in microglia, but also in hippocampal neurons. Such central anti-neuroinflammatory effects of galantamine may contribute to the observed improvement in cognition.

The MWM test and step-through test were used as robust and reliable tests that reflect hippocampal-dependent learning and memory [46–49]. We observed that LPS-induced cognitive impairments were prevented by galantamine treatment (4 mg/kg ip). Consistently in clinical studies, long-term galantamine treatment improves cognition deficits in patients with Alzheimer’s disease [50]. Some research has shown that neuroinflammatory processes are related to the pathogenesis of Alzheimer’s disease. For example, LPS levels are increased in brain samples from late-onset Alzheimer’s disease patients [10], suggesting that LPS may be involved in the cognitive impairment in Alzheimer’s disease. Overall, these suggest that galantamine may have potential to protect against LPS-induced cognition impairments in neurodegenerative diseases.

In the present study, LPS ICV injection increased the expression of the microglia marker, CD11b, in the mouse hippocampus. CD11b is one of the most important surface markers of microglia. According to reports, increased expression of CD11b corresponds to the severity of microglial activation in various neuroinflammatory diseases [51]. Furthermore, microglial activation and secretion of pro-inflammatory cytokines can induce pro-inflammatory A1 astrocytes, which are detrimental to neuronal survival, outgrowth, synaptogenesis, and phagocytosis [11]. In this study, in the hippocampus, LPS ICV injection also increased mRNA expression of GFAP (a marker of astrocyte activation) in mice, which may be attributed to activated microglia. The GFAP protein in cultured astrocytes increases approximately threefold in A1 astrocytes compared to control [11]. Therefore, the LPS injection may activate microglia and thereafter induce A1 astrocyte accumulation in the hippocampus.

Importantly, in this study, galantamine treatment prevented activation of microglia and astrocytes and improved neuroinflammation by inhibition of inflammatory signaling molecule (NF-κB p65) and cytokines (TNF-α, IL-1β and IL-6) in the hippocampus of LPS-exposed mice. Activation and translocation of NF-κB into the nucleus mediates the transcription of pro-inflammatory cytokine genes, including TNF-α, IL-1β, and IL-6 [52]. The “cholinergic anti-inflammatory pathway” is mediated by ACh binding to α7 nicotinic receptors to suppress the activation of NF-κB and inhibit the production of pro-inflammatory cytokines [53]. ACh dose-dependently decreases the release of TNF-α, IL-1β, and IL-6 from LPS-activated primary human macrophages [54]. Galantamine is an AChE inhibitor, which inhibits the AChE from dissociating ACh, thereafter increasing both the level and duration of action of the neurotransmitter ACh. Therefore, the anti-inflammatory action of galantamine may be mediated via microglia and astrocyte inhibition of the NF-κB signaling pathway. The protocol used in this study involved pretreatment of the mice with galantamine before the administration of LPS; while this is not consistent with treatment approaches in clinical settings in AD, for post-operative cognitive decline, galantamine pre-treatment may be helpful to prevent the postoperative neuroinflammation and cognitive dysfunction [55].

The synapse-associated proteins, especially presynaptic SYN and postsynaptic PSD-95, promote synaptic plasticity [56–58]. Deficits in SYN and PSD-95 correlate with cognition decline in neurodegenerative disorders, such as Alzheimer’s disease and aging [18, 19]. For example, SYN levels have been found to be decreased in the brains of oldest-aged individuals with dementia [18]. Moreover, PSD-95 levels were decreased in the brains of patients with Alzheimer’s disease cases compared with controls [19]. Aberrant dendrites and spines in the hippocampus are related to neurodegenerative disorders, such as Alzheimer’s disease [59]. In this study, the levels of presynaptic SYN and postsynaptic PSD-95 decreased in the hippocampus of LPS-exposed mice. Golgi staining showed that LPS decreased dendritic branching and dendritic spine density in the hippocampal CA1 subfield. Consistently, abnormal hippocampal neuronal plasticity has been found following neuroinflammation induced by ICV administration of LPS in rats [60]. This LPS-induced decrease in synaptic protein levels may contribute to impairment of synaptic plasticity and the learning and memory decline observed in behavioral tests, suggesting impairments of synaptic plasticity may be responsible for LPS-induced cognitive deficits. Interestingly, in our study, chronic treatment with galantamine prevented the LPS-induced reduction of SYN and PSD-95, and increased dendritic spine density, in the hippocampus. Furthermore, we also found negative correlations between CD11b or GFAP mRNA levels and synaptic proteins in the hippocampus, which suggests that the excessive activation of microglia and astrocyte may contribute to decreasing the synaptic proteins in the hippocampus. Therefore, galantamine may contribute to improved cognition in this neuroinflammatory animal model by prevention of neuroinflammation and increase of synaptic proteins in the hippocampus.

At the cellular level, this study found that microglia responded directly to LPS, while HT-22 cells responded to Mi-sup rather than LPS. In MTT assays, cell viability of HT-22 cells was not affected by LPS exposure. Previously, LPS did not increase Ca^2+^ and neither promoted neuronal apoptosis in young cultured primary hippocampal neurons expressing low levels of TLR4 [61]. Therefore, LPS may not directly impair neurons but induce neuroinflammation by firstly activating microglia. In our study, we found that cell viability of HT-22 cells was significantly decreased by exposure of Mi-sup, which produces proinflammatory cytokines and induces neural apoptosis [62]. Furthermore, in western blotting, we found that LPS directly increased NF-κB p65 level in BV-2 cells but not HT-22 cells, while the levels of NF-κB p65 in HT-22 cells were significantly increased by Mi-sup. Overall, the above results suggest that activated microglia are essential in LPS-induced neuroinflammation and mediate neuronal impairment. In the present study, galantamine prevented the increase of NF-κB p65 in BV-2 cells and HT-22 cells induced by Mi-sup. It suggests galantamine acted on the dual targets in BV-2 cells and HT-22 cells for prevention of LPS-induced neuroinflammation and neuronal impairment.

## Conclusion

This study indicates that galantamine induces cognitive improvement and anti-neuroinflammatory effects in central LPS-exposed mice. Galantamine prevented LPS-induced cognitive deficits, including spatial learning and memory as well as memory acquisition of the passive avoidance response. Galantamine also inhibited the gliosis, pro-inflammatory signaling molecules (NF-κB p65), and cytokines (IL6, IL-1β and TNF-α) and increased the synapse-associated proteins in the hippocampus of LPS-exposed mice. Furthermore, the vitro evidence showed that galantamine’s therapeutic mechanisms are not only in reducing the inflammatory response in microglia, but also in hippocampal neurons evidenced by galantamine’s ability to prevent the upregulation of NF-κB p65 in both BV-2 cells and HT-22 cells. These behavioral and neurochemical improvements in in vivo and in vitro studies suggest that galantamine may be a promising treatment to improve endotoxin-induced cognitive decline and neuroinflammation.

## Abbreviations

AChE: Acetyl-cholinesterase
CD11b: Cluster of differentiation molecule 11b
DMEM: Dulbecco’s modified Eagle’s medium
FBS: Fetal bovine serum
GFAP: Glial fibrillary acidic protein
IL-1β: Interleukin-1β
IL-6: Interleukin-6
ip: Intraperitoneal
LPS: Lipopolysaccharide
Mi-sup: Supernatant of LPS-stimulated microglia
MTT: 3-(4,5-Dimethylthiazol-2-yl)-2,5-dipheyltetrazolium bromide
MWM: Morris water maze
NF-κB: Nuclear factor-kappa B
PSD-95: Postsynaptic density protein 95
RT-PCR: Reverse transcriptase-PCR
SYN: Synaptophysin
TNF-α: Tumor necrosis factor-α
